# Optimising outputs from a validated online instrument to measure health-related quality of life (HRQL) in dogs

**DOI:** 10.1371/journal.pone.0221869

**Published:** 2019-09-18

**Authors:** Vinny Davies, Jacqueline Reid, M. Lesley Wiseman-Orr, E. Marian Scott

**Affiliations:** 1 School of Computing Science, University of Glasgow, Glasgow, Scotland, United Kingdom; 2 School of Mathematics and Statistics, University of Glasgow, Glasgow, Scotland, United Kingdom; 3 NewMetrica Ltd, Glasgow, Scotland, United Kingdom; Colorado State University, UNITED STATES

## Abstract

Measurement of health-related quality of life (HRQL) is becoming increasingly valuable within veterinary preventative health care and chronic disease management, as well as in outcomes research. Initial reliability and validation of a 22 item shortened version of VetMetrica (VM), structured questionnaire instrument to measure HRQL in dogs via a mobile application was reported previously. Meaningful interpretation and presentation of the 4 domain scores comprising the HRQL profile generated by VM is key to its successful use in clinical practice and research. Study one describes transformation of domain scores from 0–6 to 0–100 and normalisation of these based on the healthy canine population in two age ranges, such that a score of 50 on a 0–100 scale represents the score for the age-related average healthy dog, and establishment of a threshold to assess domain-specific health status for individual dogs. This provides the clinician with a simple method of ascertaining the health status of an individual dog relative to the average healthy population in the same age group (norm-based scoring). Study two determines the minimum important difference (MID) in domain scores which represents the smallest improvement in score that is meaningful to the dog owner, thus providing the clinician with a means of recognising what is likely to be a significant improvement in scores for an individual dog over time. Visual representation of these guidelines for the purpose of interpreting VM profile scores is presented using case studies.

## Introduction

Health-related quality of life (HRQL) has been the cornerstone of outcomes research for three decades and increasingly is an important focus in human health measurement where instruments are developed to measure chronic disease in people through its impact on HRQL. An important goal of evidence-based medicine has been to incorporate patient reported HRQL scales to better assess clinical outcomes [1]. Instruments to measure HRQL can be disease specific or they can be generic [2–7]. Generic HRQL measures are intended for general use and can be used in healthy as well as sick populations. They have an advantage over disease-specific measures in that they may be the only option when co-morbidities exist, as is often the case in older patients. Generic instruments can generate a single item score, or they can be profile measures. Profile measures typically reflect an individual's current health status on multiple domains of HRQL and assign a score to each domain, facilitating the detection of different effects on different dimensions of HRQL.

Interpretability is a key feature of a useful measurement scale, but currently there is no consensus as to how HRQL scores should be presented optimally for ease of interpretation by the instrument user. Users must be able readily to understand what an individual score generated by an instrument means, for example by comparison with a healthy population. They also need to know when any change in those scores is meaningful: is the treatment really working or the disease really worsening over time? This is currently an important research focus in the medical field [8], where according to these authors “the choice of what constitutes an important difference in a HRQL score will influence judgements about the success of a health care intervention, the required sample size of clinical studies, and the design of these studies.”

The development of HRQL instruments is an expanding area in veterinary science and there is increasing awareness of the value of these tools to measure wellness within a model of veterinary preventative healthcare and to measure the impact of chronic disease and its treatment [9]. Accordingly, interpretability is equally as important in the veterinary as well as the medical field and is likely to become more so as more instruments are developed for use in different populations and contexts.

Previously we have reported the development, validation and reliability of a web–based 46-item generic HRQL structured questionnaire instrument, VetMetrica (www.vetmetrica.com)[10–12], and its shortening to 22 items for the purpose of improving its utility and facilitating its presentation via a smartphone app [9]. The app comprises 22 questions for the pet owner which can be completed in around 5 minutes with automatic and instantaneous computation and reporting of scores in 4 domains of HRQL–Energetic/Enthusiastic (E/E), Happy/Content (H/C), Active/Comfortable (A/C), Calm/Relaxed (C/R). In addition to VetMetrica, only two generic methods to measure HRQL in dogs have been published, but one was shown not to distinguish healthy from sick dogs [13,14] and the other [15] was restricted to use in healthy dogs. In contrast, VetMetrica was developed using information from owners of sick and healthy dogs [10] and had been shown to distinguish between these groups [11,12,9]

A variety of methods have been described to improve interpretability of HRQL instruments including facilitating the meaningful comparison of the scores between instruments that are measuring the same thing but on different metrics that were derived during development [16]. One common approach to equating scores reported using different metrics is to transform the raw scores to normalised scores (T-scores). This ‘transforms’ each scale to have a mean of 50 and a standard deviation (SD) of 10, rendering them comparable [17].

Another approach to facilitate interpretation relates the HRQL scores to those of a specific population, facilitating judgment as to whether an observed score is typical of what would be expected for that population (norm-based scoring). Norm-based comparisons can be related to the general population, to sub-populations with shared demographics such as age or gender or to a population with a particular disease [18].

Additionally, Schunemann, Akl and Guyatt [8] describe how clinicians can use intuitive thresholds to interpret HRQL scores, by referring to an absolute score above which a patient will be deemed to have improved with treatment or by associating a change in score with improvement or deterioration. An extension of this empirical approach is to quantify a score change by calculating the minimum important difference (MID), defined as “the smallest difference in score in the outcome of interest that informed patients or informed proxies perceive as important, either beneficial or harmful, and which would lead the patient or clinician to consider a change in the management” [19].

The MID can be established with distributional and/or anchor-based techniques. Distribution-based methods rely on the statistical properties of the instrument with no reference to an external impression of change and include effect size [20], normalised response mean [21] and the modified normalised response mean [22]. Conversely, anchor-based techniques use an external criterion such as the patient’s perspective of meaningful improvement or worsening to identify the change on the HRQL scale that corresponds to the MID [23]. However, such global measures of change are strongly influenced by the context in which they are applied and subject to much variability making reliance on these problematic [16].

A more objective approach uses receiver operating characteristic (ROC) curves which are commonly used to determine the accuracy of diagnostic tests. In order to use ROC curves, we calculate the characteristics of the test, namely sensitivity and specificity, which describe how well the test discriminates between two groups. For example, sensitivity describes how well a test identifies those with a particular disease (true positive) and specificity describes how well it correctly identifies those without that disease (true negative). The ROC is then a plot of trade-offs between sensitivity and specificity and is used to choose a cut-off (threshold value) above which cases are classified as positive while cases with scores below that cut-off are classified as negative. Each cut-off value gives a different combination of sensitivity and specificity. The ROC curve plots true positive rate (sensitivity) against false positive rate (1 –specificity), giving a picture of the whole spectrum of such trade-offs and its shape is used to make decisions about the most appropriate cut-off. A test with perfect discrimination (no overlap in the diseased and non-diseased distributions: no false positives or false negatives) has a ROC curve that passes through the upper left corner of the ROC space (graph), providing 100% sensitivity and 100% specificity, but this is very rare. Therefore, the closer the ROC curve is to the upper left corner of the graph, the higher the overall accuracy of the test [24].

Deyo and Centor [25] suggested that scales could be viewed as “diagnostic tests” for discriminating between patients that had improved and those that had not and that ROC curves could be used to describe a scale's ability to detect improvement. Accordingly, in the context of this study, the owner’s impression of change (improved or unchanged) and the corresponding change in HRQL score were used to calculate a series of sensitivity and 1 –specificity value pairs, which then made up the points on the ROC curve. Each point on the ROC curve can be translated back to a value: in this case a change in score. A point on the curve could then be chosen as the MID with due regard given to the consequences for sensitivity and specificity, taking account of the clinical implications of that choice.

The aims of this research were firstly to normalise the scores generated by the VetMetrica instrument for dogs, where 50 would represent the average score for the age related healthy dog population and use these to establish a HRQL threshold score which would assist in determining the health status of individual dogs (healthy vs sick) (Study 1) and secondly to determine the MID for a general population of dogs (Study 2).

## Materials and methods

All data were retrospective having been collected in a variety of previous studies. All studies were approved by the Ethics Committee of the University of Glasgow Veterinary School and owners gave informed consent for participation in these studies. All analyses for studies 1 and 2 were carried out using the statistical software R (https://www.r-project.org/).

## Study 1: Normalising the domain scores based on the healthy population and establishing thresholds to assess domain-specific health status for individual dogs

### Data

HRQL data generated by owners of 159 healthy dogs that had participated in field tests for validation of a 109-item prototype paper-based instrument [11], the 46-item VetMetrica instrument [12] and the 22-item shortened instrument [9] were used for the normalisation process. For the determination of thresholds, data generated by 263 owners of sick dogs from the same sources were also included. Where owners had completed the 109-item prototype and the 46-item instrument, the 22 items comprising the shortened instrument were extracted and application of the 22 item scoring algorithm generated raw scores (0–6) in the 4 domains of HRQL–E/E, H/C, A/C and C/R. Data were grouped according to whether the dog was classified as young (0–7 years) and heathy (n = 129), young and sick (n = 41), old (≥8 years) and healthy (n = 30) or old and sick (n = 222).

### Methods

#### Normalisation process using healthy dogs

Step 1: Transformation of 0–6 scores to a continuous scale, using a logit transformation.

To allow the use of a logit transformation, HRQL scores (d) on the scale of 0–6 were converted to lie between 0 and 1, excluding exact 0 and 1 values. This was achieved by adding an arbitrary value, 0.1, (giving *d* in the equation below) at each end of the scale and dividing by 6.2 (the maximum score as follows:

$$d^{\prime }=\frac{d+0.1}{6.2}.$$

Thereafter these converted scores were *logit* transformed to the continuous real scale (values between very large negative and very large positive values) as follows:

$$d^{\prime \prime }=logit(d^{\prime })=\log\left(\frac{d^{\prime }}{1-d^{\prime }}\right).$$

The transformation to a *logit* scale puts the measurement on a continuous scale., which is standard statistical practise in the testing literature [26].

Step 2: Following transformation to a continuous distribution, T-scores were calculated separately for each group (4 HRQL domain scores for young dogs and 4 for old dogs) based on the sample means (*μ*), and sample SDs (*σ*), of the scores as follows:

$$T=\frac{d^{\prime \prime }-\mu }{\sigma }.$$

The T-scores have an assumed mean 0 and standard deviation 1. Finally, T-scores were scaled by multiplying them by 10 and adding 50, thus providing easily interpretable scores on a common scale where a score of 50 represented the healthy population norm for a given age group and HRQL domain:

S=10×T+50.

In combination, these transformations produce scores that, as desired, are comparable across different age groups and HRQL domains. The transformed scores will not, however, follow a normal (or Gaussian) distribution due to a ceiling affect observed within our data. The ceiling effect is a result of the large number of healthy dogs achieving a maximum score of 6. Various approaches (both parametric and non-parametric) have been proposed for dealing with this, e.g. [27,28], but none of these work effectively with our heavily right skewed data. As these approaches are non-optimal in this case, we have chosen to use the standard approach taken in the statistical testing and normalisation literature, as described above, in order to keep our approach easily understandable and interpretable [26,29].

#### Establishing thresholds between healthy and sick dogs

To investigate the separation between the healthy and sick dog populations, boxplots of the normalised HRQL domain scores (4 for young dogs and 4 for old dogs) were constructed as well as a density plot of the theoretical distribution of the healthy dog populations of both young and old dogs. Thresholds were determined using the deciles of the theoretical density of the healthy dog population. While the true density will not follow the theoretical density exactly, using the theoretical properties will allow us to choose a threshold which is consistent across all ages and HRQL domains, with each age and domain combination having the same mean and standard deviation. Once the theoretical density has been created, boxplots were then visually examined for each group and domain combination in order to identify a consistent threshold which best separated the healthy and sick populations.

#### The practical application of normalisation of scores and threshold setting

Three examples were drawn from studies conducted with the instrument. Example 1 illustrates the difference between the HRQL profile of a group of dogs treated with regenerative medicine using raw and normalised scores. Example 2 is a cross-sectional study of 2 groups of dogs with ocular conditions and the third is of an individual dog whose HRQL was recorded over time.

### Results

#### Normalisation process

A score of 50 on the normalised/standardised scale represents the healthy population norm for a given age group and HRQL domain.

#### Establishing thresholds between healthy and sick dogs

**Fig 1:**
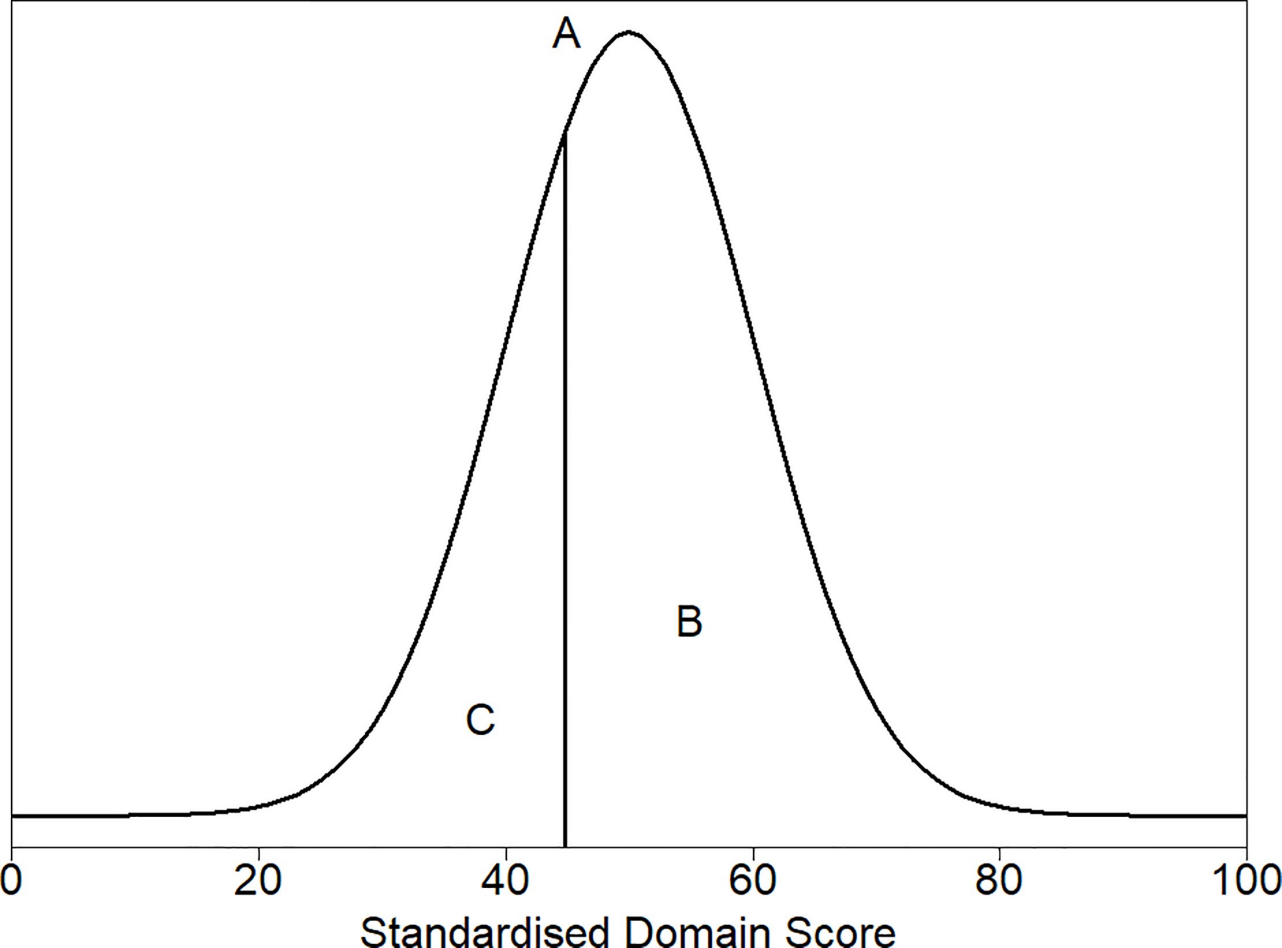
Density plot of the theoretical distribution of the healthy dog populations of both young and old dogs. Marked on the plot is (A) a vertical line denoting the threshold for dividing healthy and sick dogs, the 30^th^ percentile (a score of 44.8). Above this threshold (to the right on the plot) are 70% of the healthy dog population predicted to be healthy, the area marked by B. Below the threshold (to the left on the plot) are 30% of the healthy dog population predicted to be sick, the area marked by C.

Fig 1 shows the theoretical (normal) distribution of the normalised scores of the healthy population of dogs, old and young combined. The 30^th^ percentile, which is the value above which scores for 70% of the healthy population will lie, was chosen as the threshold for both young and old dogs, corresponding to a normalised score of 44.8.

**Fig 2:**
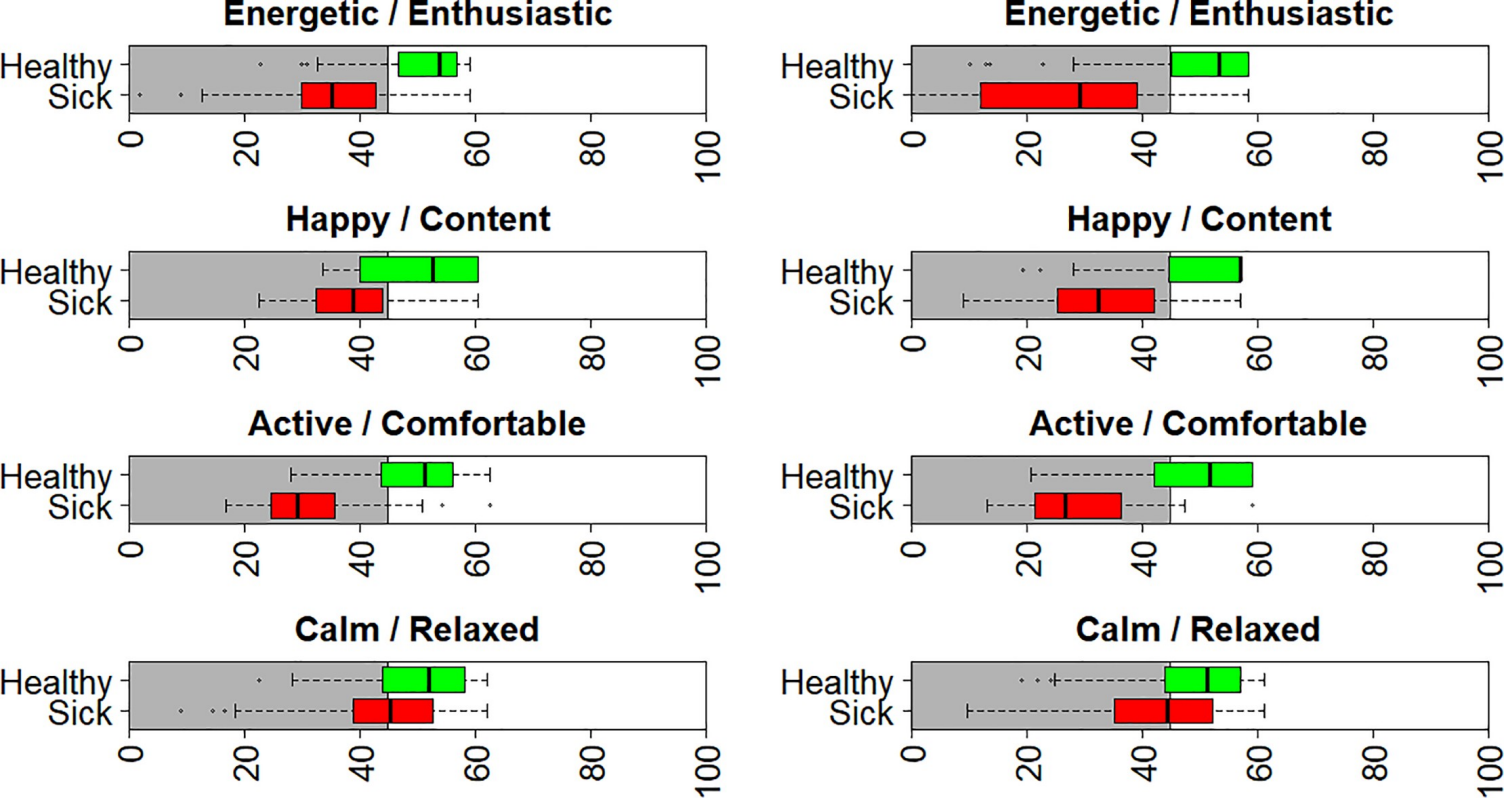
Boxplots of the normalised scores for young (left column) and old (right column) dogs. Boxplots are shown separately for healthy and sick dogs. The threshold is 44.8, with dogs above this point estimated to be healthy since 70% of healthy dogs lie above this threshold. The density plot explaining the choice of the threshold is given in Fig 1.

Fig 2 shows box plots of the resulting distributions of the normalised HRQL scores for young (left column) and old (right column) dogs. In each case, the dogs are divided into sick and healthy, indicated by the red and green box plots respectively. Scores overlap between healthy and sick groups in all 4 domains, although there is a separation of the interquartile ranges (25–75 percentile) in E/E and A/C for young dogs and E/E, H/C and A/C for old dogs, but no separation in either group for C/R.

#### Practical application—Case examples

**Fig 3:**
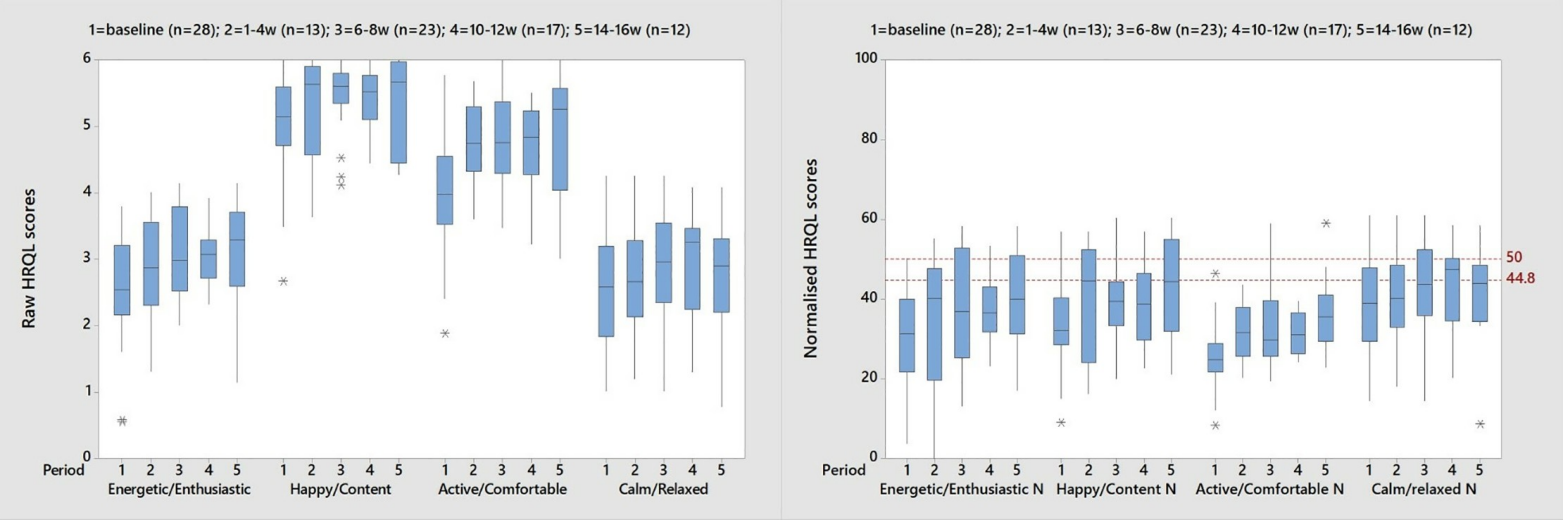
A comparison of raw scores and normalised scores from a study of dogs with osteoarthritis and associated soft issue pathologies treated with regenerative medicine. Raw HRQL scores (left) and Normalised HRQL scores (right). 50 represents the score for the average healthy dog and 44.8 is the threshold above which 70% of healthy dogs will score.

Fig 3 compares the HRQL profile of a group of osteoarthritic dogs with associated soft tissue pathologies treated with stem cells and platelet-rich plasma (PRP), using the raw scores and the normalised scores. The raw scores reveal broad improvement over time in scores on all domains, but any further interpretation is difficult. (Note the possible ceiling effects in two domains, namely H/C and A/C, which is when an instrument fails to capture variability above certain values). However, the normalised scores for each domain are much more readily interpretable, both in relation to each other and to the chosen common threshold value of 44.8.

**Fig 4:**
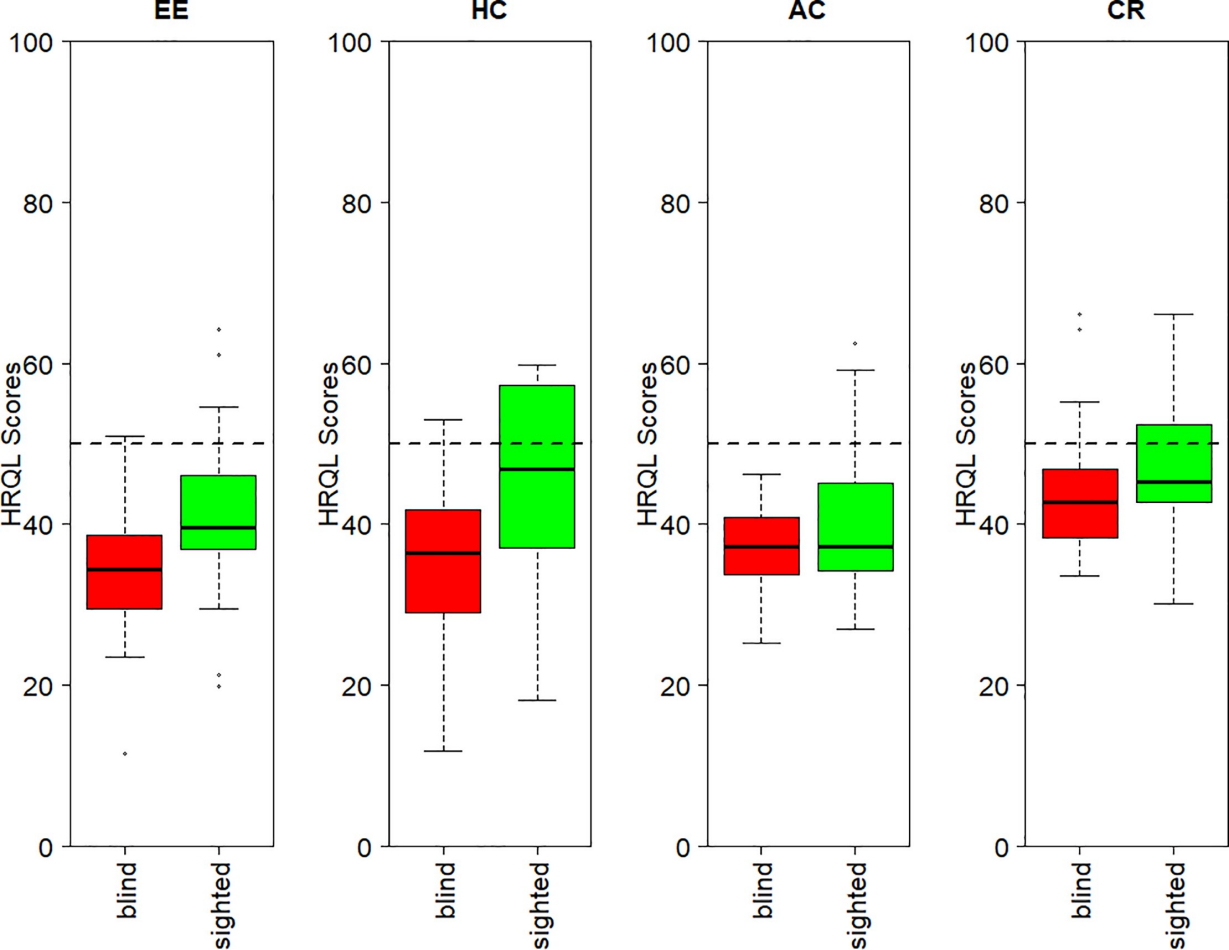
Boxplots of HRQL scores in 4 domains for 2 groups of dogs with ocular disease, one blind and the other with functional vision. A score of 50 represents that of the average healthy dog according to age.

**Fig 5:**
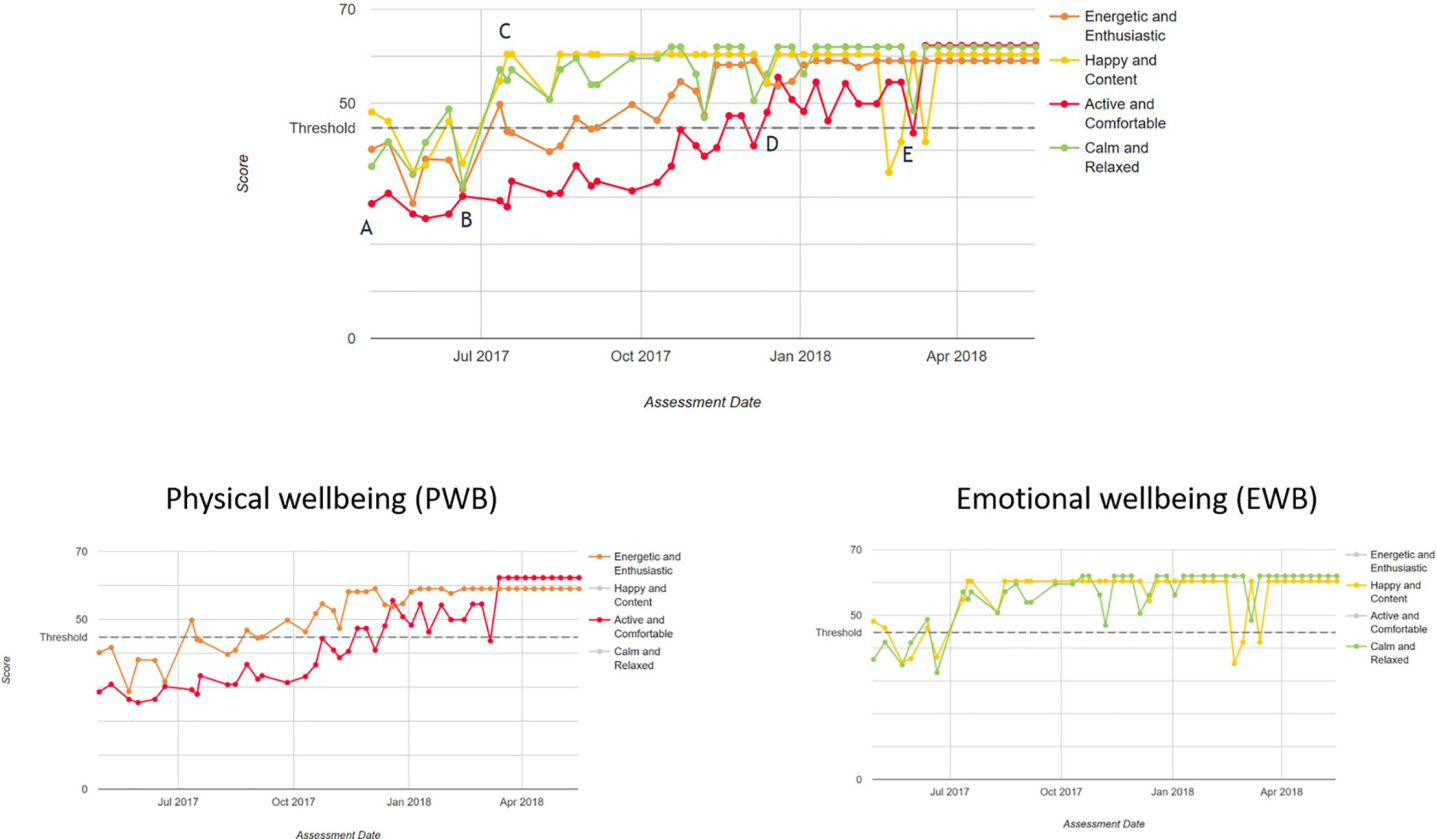
HRQL scores in 4 domains for an 11 year old Sharpei dog with osteoarthritis (OA) and inflammatory bowel disease (IBD), treated over 11 months. A–B treatment with acupuncture; C onwards, lifestyle changes, hydrotherapy, laser; E flare-up of IBD.

Figs 4 and 5 illustrate how HRQL scores can be interpreted and used to discriminate between groups at a single point in time and for the evaluation of change in an individual over time. Fig 4 shows the HRQL profiles of 2 groups of dogs presented to a specialised eye clinic for a variety of ocular conditions only (no comorbidities were present). Dogs in group 1 (n = 25) were blind and those in group 2 (n = 43) had functional vision. The median HRQL scores for both groups of dogs were below the healthy dog average for all domains indicating that those ocular conditions present did have an impact on the dogs’ HRQL. The differences between the groups were not significantly different at the 5% significance level for A/C and C/R (p = 0.26 and p = 0.06 respectively), but there was a statistically significant difference in E/E (p = 0.002) and H/C (p = 0.002).

Fig 5 is the HRQL profile for an 11-year-old Shar Pei dog with osteoarthritis (OA) and inflammatory bowel disease (IBD), recorded over a period of 11 months. Scores for each domain are colour coded and the threshold score (44.8), above which 70% of healthy dogs will lie, is shown as the dotted line. In addition to the full profile, the 4 domains are shown subdivided into 2 groups, one depicting domain scores related to physical wellbeing (PWB) and the other to emotional wellbeing (EWB). At referral to a pain and rehabilitation unit (point A) all domain scores were below 50 (average healthy dog of that age group) and moreover, with the exception of H/C, these fell below the threshold above which lies 70% of healthy dogs. Between time points A and B, the dog had acupuncture which produced an initial improvement, but treatment ceased due to poor compliance of the dog. Thereafter hydrotherapy and lifestyle management formed the basis of treatment and by point C scores for E/E, H/C and C/R had risen above the threshold, with a continuing trend for improvement. Active/comfortable showed a much slower improvement, but by point D scores exceeded the threshold. The deterioration in scores at point E corresponded to a flare up of the dog’s IBD. The maximum improvement in emotional wellbeing peaked at approximately 1 month whereas in physical wellbeing that point was not reached until month 11.

## Study 2: Determining the MID of a general population of dogs using normalised HRQL scores

### Data

Data from healthy and sick dogs obtained during the 22-item field test as before and from an unpublished clinical trial were used. Owners had completed at least two questionnaires and simultaneously recorded whether their dog’s health status had improved, stayed the same or worsened since the previous assessment. Only the first and second assessments were used for the analysis and raw domain scores were normalised to the healthy population as described in study 1.

Data from 11 dogs classified as worsened were removed on the basis of the small sample size, leaving 212 dogs, 67 assessed as having improved and 145 that had not changed in health status. The unchanged group contained healthy and sick dogs comprising both age groups.

### Methods

#### Anchor based method using ROC curves

Using data from both healthy (n = 113) and sick (n = 99) dogs, the difference in the normalised scores in each HRQL domain between the 1^st^ and 2^nd^ assessment was calculated for each dog and these follow an approximately normal distribution. The resulting mid-points between these change values (possible MIDs), the change values themselves and the corresponding owner impression of change (unchanged or improved) were input as variables into the statistical software, to create sensitivity and specificity pairs for each change value. There were insufficient cases where dogs were deemed to have deteriorated for inclusion in this analysis. Sensitivity was plotted against 1 –specificity, to create ROC curves for all four domains. Following construction of these ROC curves the most appropriate MID value from all 212 possible values (113 healthy and 99 sick dogs) was determined on the basis that it is equally important to correctly identify a dog that has improved in health as one that is unchanged. This does not guarantee that sensitivity and specificity are going to be approximately equal in any given domain, one may be much higher than the other based on the distribution of the scores, but simply means they are prioritised equally in the method used to determine the MID. After completing this for each individual domain (ROCdomains), the same method was applied to generate a MID that was consistent across all HRQL domains (ROCconsistent). This was done by combining all the data used for each individual HRQL domain into a combined group and creating a single ROC curve, as opposed to multiple ROC curves as previously and determining the MID on that curve on the same basis as before. The clinical implications (in terms of sensitivity vs specificity) of applying the MIDconsistent to each domain were then carefully considered.

### Results

**Fig 6:**
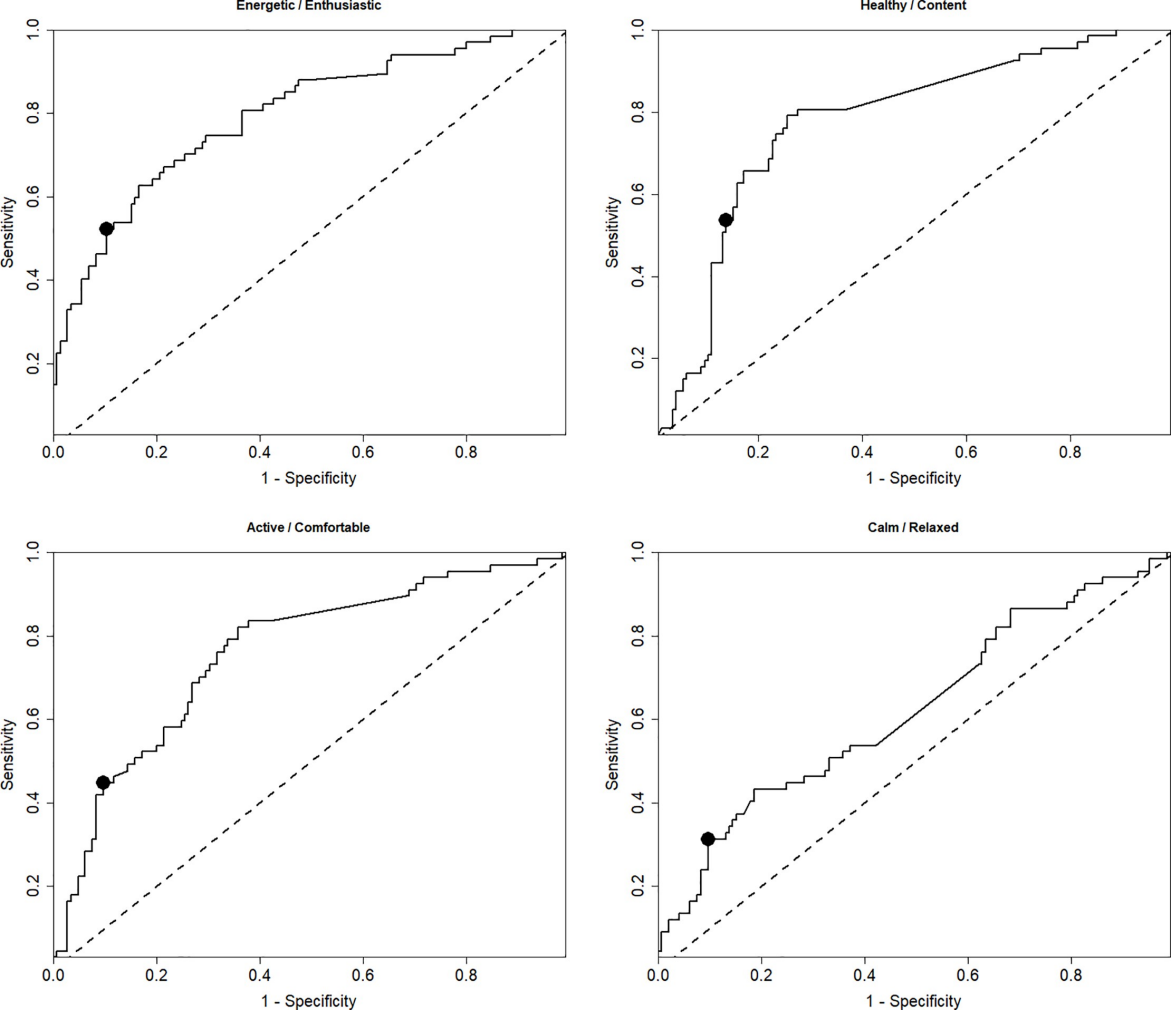
ROC curves show all possible MIDs with the corresponding sensitivities and 1 –specificities. ROC curves are shown for (A) Energetic / Enthusiastic, (B) Healthy / Content, (C) Active / Comfortable and (D) Calm / Relaxed. The MIDconsistent cut-off is plotted as a large point on each ROC curve.

Fig 6 shows ROC curves for each HRQL domain respectively. In each case the ROC curves show each possible trade-off of sensitivity and specificity for that HRQL domain. The results for the ROCdomains method (MIDdomains) are given in Table 1, along with those for the ROCconsistent (MIDconsistent). Table 1 gives the sensitivity, specificity, classification accuracy (the number of correct classifications) and the Area Under the ROC curve (AUC) for each of the MIDdomains. The MIDconsistent change value which resulted from the analysis using a single ROC based on combined data was 7.7. This location is shown as large points on the ROC curves for each domain shown in Fig 6. The clinical implications in terms of sensitivity vs specificity of applying the MIDconsistent of 7.7 to each domain was carefully considered and determined to be appropriate.

**Table 1:** Table comparing the sensitivities, specificities, classification accuracy and AUC of each MID for each HRQL domain: ages have been combined but normalised separately.

HRQL domain	MID type	Score Change (MID)	Sensitivity	Specificity	Classification Accuracy	AUC
**E/E**	MIDdomain	*7*.*6*	*0*.*52*	*0*.*90*	*0*.*78*	*0*.*80*
	MIDconsistent	*7*.*7*	*0*.*52*	*0*.*90*	*0*.*78*	*0*.*80*
**H/C**	MIDdomain	*6*.*4*	*0*.*62*	*0*.*84*	*0*.*77*	*0*.*77*
	MIDconsistent	*7*.*7*	*0*.*54*	*0*.*86*	*0*.*76*	*0*.*77*
**A/C**	MIDdomain	*7*.*7*	*0*.*45*	*0*.*90*	*0*.*76*	*0*.*76*
	MIDconsistent	*7*.*7*	*0*.*45*	*0*.*90*	*0*.*76*	*0*.*76*
**C/R**	MIDdomain	*8*.*1*	*0*.*31*	*0*.*90*	*0*.*72*	*0*.*62*
	MIDconsistent	*7*.*7*	*0*.*31*	*0*.*90*	*0*.*72*	*0*.*62*

**Fig 7:**
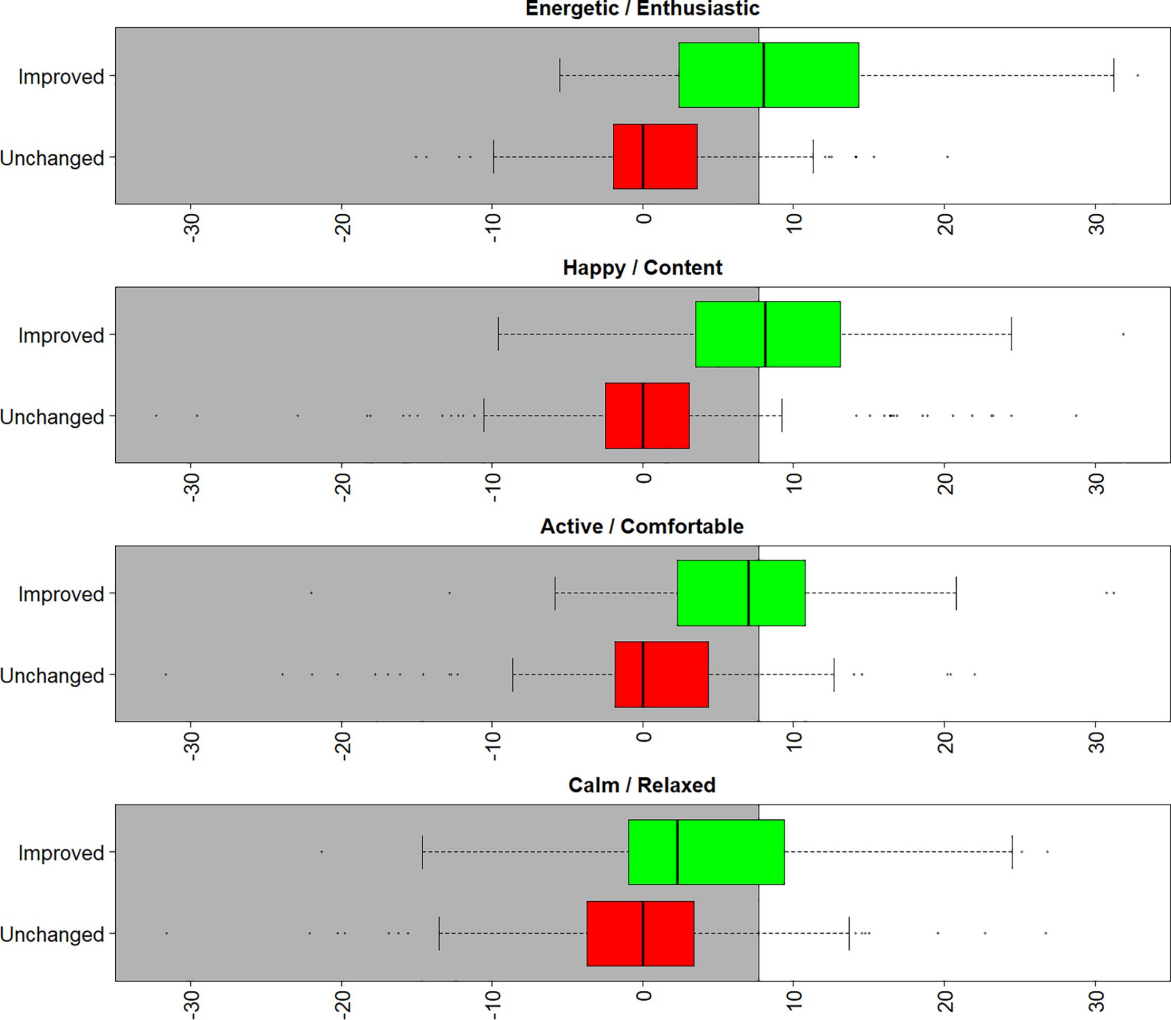
Box plots of the change in the 4 normalised HRQL scores over multiple observations of the same dogs. Differences in the scores are shown as boxplots, where green indicated a dog was reported to have improved and red where no change was observed. The grey area shows where a dog would be classified by MIDconsistent to be unchanged and white shows where the classification would be of improvement: the cut-off for this is 7.7 (Table 1).

Fig 7 shows boxplots of the change in HRQL domain scores for dogs whose owners report that they have improved or are unchanged, as indicated by green and red boxplots respectively. On each boxplot, the area below the MIDconsistent is indicated by a shaded area. Scores overlap between improved and unchanged dogs in all HRQL domains, although in the H/C domain a demarcation can be seen between the interquartile ranges (25–75 percentile). Reasonable division between the E/E and A/C can however still be seen, although there is little differentiation between the improved and unchanged dogs in C/R.

## Discussion

This paper describes how three individual strategies, reported to enhance the interpretability of human HRQL instruments, namely normalisation of scores, thresholds to provide guidance regarding state of health, and MID, have been used to improve the interpretability of VetMetrica, a HRQL instrument for dogs.

In human medicine normative reference data on health measurement scales are generally presented in tabular form by age and gender from a general population sample [30]. However, an alternative approach was taken by The Medical Outcomes Study Short-Form 36-Item Health Survey (SF-36) for people, a widely used generic instrument capable of measuring the HRQL of individuals with different diseases or health conditions, whereby a “built in” reference has been developed. Norm-based scoring algorithms transform the raw scores so that final scores are reported on a continuous scale where the mean of 50 represents the US population average and the standard deviation is 10 [31]. The SF-36 generates scale scores for eight domains of HRQL and two summary scores–a physical component summary (PCS) and a mental component summary (MCS). Since two of the VetMetrica domains are associated with physical wellbeing and two with emotional wellbeing, it may in future be possible to evaluate the measurement properties of summary scores for such components–physical wellbeing and emotional wellbeing. Fig 5 demonstrates the temporal difference in improvement between physical and emotional components of the HRQL profile. To the authors’ knowledge this is the first time such an observation has been reported in relation to clinical improvement in dogs. Normative scoring makes it easier to interpret and compare scale scores which have different average values and standard deviations on the traditional 0–100 metric: now for all scales, scores above 50 are better than average and those below are worse compared to the healthy population [32].

As a generic instrument generating a profile of scores in four domains of HRQL, VetMetrica is very similar to the SF-36 and accordingly it was decided to adopt the same approach of building in norms to the scoring algorithms. However, in contrast to the SF-36, VetMetrica scores were normalised to the healthy dog rather than the general population, for two reasons. Firstly because of the difficulties of obtaining a sample of dogs which would be representative of the general population, and secondly because scores derived from a specific general population like the SF-36 have been criticised for their lack of generalisability across different geographical and cultural populations [33]. In the authors’ opinion the generalisability of HRQL scores in the healthy dog population, at least in the western world, is unlikely to be affected to the same extent by population differences, thus justifying their choice of healthy population norms.

In addition to the healthy dog comparison, the normalisation process presented here also incorporated age. This is in contrast to the SF-36 where norms other than the general population are not included in the scoring algorithms but are presented separately in a standard tabular format. Age norms are commonly used in human medicine where they are generally combined with gender, but for veterinary populations, gender-based norms are inappropriate because the majority of dogs are neutered by the age of 1 year. VetMetrica scores were normalised to two age groups, young (0–7 years) and old (8 years and over). The authors recognise that 0–7 years is a very broad range where it is perhaps unrealistic to class the HRQL of a one-year-old dog with that of a seven-year-old, but there were insufficient data to separate young and middle-aged dogs into two groups. This limitation will be addressed as more data become available.

In addition to normalising the scores so that 50 represents the average healthy dog in the subject’s age group, a threshold score was derived to provide additional information regarding health status. Ideally that threshold would separate healthy from sick dogs, but there was considerable overlap in the scores between healthy and sick dogs. This is not surprising given the broad age ranges, the heterogeneity of disease and the likely influence of breed and of other factors, unrelated to health, on quality of life. Accordingly, and to aid interpretability, the threshold was set at 44.8 the 30th percentile, for all 4 HRQL domains for both old and young dogs, such that 70% of healthy dogs will score above that threshold.

One potential concern with the normalisation process is that it does not deal with the ceiling effects which occur in some HRQL domains. Ceiling effects occur when scores reach the maximum score, in this case a result of a large number of healthy dogs which we expect to score very highly. Several statistical methods, both parametric and non-parametric, have been proposed to deal with ceiling effects [27,28]. However, due to the large number of dogs that achieve a maximum score in some of our HRQL domains, these will not be effective at correcting the skewness of our data. We have therefore chosen to continue to follow the standard statistical practise of transforming data to a continuous scale [26], and then calculating norm-based scores which are common in the literature and comparable across all the domains [29]. It is also important to note that the ceiling effect does not occur when determining the MID, where the difference in normalised scores is approximately normally distributed.

Fig 3 compares the raw score profile for a group of dogs undergoing regenerative therapy for OA with the profile once the scores are normalised, demonstrating the relative ease of interpretation of normalised scores. For example, when compared with the average healthy dog in the appropriate age group it is clear A/C is affected more than the other domains and although all domains show improvement over time scores in A/C remain relatively depressed throughout treatment. Furthermore, the two case studies presented demonstrate how the normalised scores and health status threshold provide the veterinary surgeon with an immediate visual interpretation of individual or group scores relative to health status over time.

However, some change in quality of life is to be expected over time since it may be influenced by a wide range of factors, not just health state. Accordingly, it is important to distinguish clinically significant change from ‘noise’. For example, it is important to be able to advise owners of healthy dogs that a certain amount of change is within normal parameters, and not likely to indicate any change in health state. Conversely, in the case of a sick dog the clinician needs to know when an improvement in scores can be interpreted as a positive effect of treatment. This is the function of the MID. Equally important would be the ability to determine if a deterioration in scores represented a meaningful change, but unfortunately there were insufficient data to investigate this. Given the fact that clinical data confirming a change in health status were not available, and owners are perhaps reluctant to admit to a deterioration in their dog, it is difficult to ascertain whether some ‘unchanged’ dogs were in fact worse. An alternative explanation might be that owners habituate to their dog’s health state and actually believe that they are unchanged when in fact their condition has deteriorated. The authors accept that this is a significant limitation to the use of the scale which will be addressed as more data become available.

Each point along the ROC curve comes from an algorithm that includes a varying amount of change, whether dogs’ scores have changed or not, and whether owners reported change or not. Each point represents a trade-off between sensitivity and specificity so that, along the curve created, the point at the top right of the curve will classify all dogs as improved and that at the bottom left of the curve points will classify all dogs as unchanged. The curve can then be used to choose a point (each which relates to an amount of change in scores) that optimises sensitivity and specificity in terms of correctly (according to owners’ assessment) classifying a dog as improved or unchanged.

Sensitivity is the extent to which true positives are not missed/overlooked (so few false negatives) and specificity is the extent to which positives really represent the condition of interest (few false positives). The ideal situation would be to have the sensitivity and specificity as close to 1 as possible simultaneously, but in general this is not possible. Therefore, the final choice of MID seeks a balance between sensitivity and specificity. In determining a MID for patients with chronic conditions such as low back pain, sensitivity and specificity are given equal importance [34]. Similarly, epidemiologists agree that sensitivity and specificity should be valued equally, although they may use different approaches to calculate the optimum MID cut-point using ROC curves [35, 36]. Normally, the point chosen on a ROC curve will be the one that is highest and furthest left on the curve as we have done here, maximising both sensitivity and specificity, although this approach can be varied according to clinical relevance.

In this study, we firstly established MIDs separately for each domain, MIDdomains, resulting in reasonable performance (Table 1) in terms of sensitivity, specificity and classification accuracy for E/E, H/C and A/C, with slightly poorer performance for C/R which has less predictive power. We then took an additional step and chose a MID that could be applied consistently across all four domains, MIDconsistent. This was done to make interpretation of the scores as simple as possible for users. The MID consistent provided the same degree of sensitivity and specificity as MIDdomains for E/E, A/C and C/R. H/C had a slightly increased specificity and slightly reduced sensitivity, resulting in a very slightly reduced classification accuracy. Although the lower sensitivity than specificity provided by both MIDdomains and MIDconsistent means that a number of dogs will be diagnosed as having not changed when in fact they have improved, on balance this makes clinical sense. It errs on the side of caution in terms of not diagnosing dogs as improved when they have not. Overall, the results of Table 1 represent useful levels of accuracy providing that those using the instrument and interpreting its output understand the limits of its accuracy and use their clinical judgement accordingly.

In contrast to disease-specific instruments, generic instruments such as the SF-36 and VetMetrica are designed to provide a summary of HRQL which can change for a variety of reasons including the burden of chronic disease in general, hence the use of dogs with a wide range of chronic diseases to calculate the MID in this study. However, these generic tools can be used satisfactorily in specific disease states although they may be less sensitive than disease specific tools. For example, the use of the SF-36 has been reported in many disease specific populations, including Crohn’s Disease [37], osteoarthritis [38], cardiac disease [39] and asthma [40]. The MID varies considerably between these conditions and this highlights the fact that, like validity, the MID is not an inherent property of the scale, but a feature of the scale as it is used in a particular clinical context. Accordingly, the MID of 7.7 calculated in this study for a general population may not apply when the scale is used in specific disease populations.

It is interesting to note that in the boxplots used to determine the health status threshold, there was more overlap in normalised scores between healthy and sick dogs in C/R than in the other three domains. Similarly, there was less distinction between dogs whose health status, according to the owner, had changed compared to those who had not in C/R compared with the other domains. This discrepancy between domains has been noted before [9] and has been attributed to the fact that this domain may to some extent reflect relatively stable personality traits, making it more resistant than other domains to change with ill health.

Small sample sizes limited the scope of these studies. While the available data were considered adequate for the normalisation to the average healthy dog, the age norm was limited to two age groups which may be considered too broad, especially in the ‘young’ group which includes middle-aged dogs. Similarly, sufficient data were not available to incorporate breed norms, which the authors consider likely to be an important addition for dogs. Intuitively, with the possible exception of H/C and C/R, the remaining domain scores are likely to reflect to some extent differences between the lives commonly led by different breeds, such as a relatively sedentary toy breed and that of a more active working breed. Investigation of such differences will form the basis of future refinement of the tool when such data are available, as will the reporting of additional age norms. Clearly, a useful HRQL instrument will also discriminate between subjects that decline as well as improve. However, in this study there were too few dogs whose owners considered that they had worsened to make the calculation of a MID for deterioration possible. Nevertheless, it should be noted that HRQL instrument development is an iterative process whereby it is not uncommon for existing tools to undergo a continual process of refinement to accommodate new populations and contexts in which they are to be used. [16]

Despite these limitations, the authors consider that this work contributes substantially to the body of knowledge regarding the interpretation of scores provided by HRQL instruments in veterinary science. To their knowledge, at the time of submission of this paper, there are no published reports of the processes described here for generic or disease specific HRQL instruments for use in animals.

## Supporting information

S1 File Study 1 data for old dogs. (CSV)

S2 File Study 1 data for young dogs. (CSV)

S3 File Study 2 data. (CSV)
